# Aging in Place or Institutionalization? A Multiscale Analysis of Independent-Living Older Adults From Four Large Cities in China’s Yangtze River Delta

**DOI:** 10.1093/geroni/igad014

**Published:** 2023-02-21

**Authors:** Zhongyu He, Cailing Jiang

**Affiliations:** School of Architecture and Urban Planning, Nanjing University, Nanjing, China; School of Architecture and Urban Planning, Nanjing University, Nanjing, China

**Keywords:** Age-friendly, Community-built environment, Health, Preference for place to grow older, Social environment

## Abstract

**Background and Objectives:**

Aging in place (AIP) has been adopted as a key strategy to cope with the global public health challenge posed by population aging. The current study aimed to understand the association between older adult’s AIP preference and various social and physical environmental factors at different scales.

**Research Design and Methods:**

Following the ecological model of aging, this paper conducted a questionnaire survey of 827 independent-living older adults (60 years old and above) from four big cities in China’s Yangtze River Delta region and employed a structural equation modeling method for analysis.

**Results:**

Older adults from more developed cities exhibited a stronger preference for AIP than those from less developed cities. Individual characteristics, mental health, and physical health had a direct impact on AIP preference, whereas the effect of the community social environment was not significant. The perceived and objectively measured community-built environment indirectly affected AIP preference via mediation and chain effects.

**Discussion and Implications:**

Complex paths affecting AIP preference were identified. At the city level, the social environment had a stronger influence than the physical environment on AIP, and the opposite pattern was observed at the community level. Mental health and physical health had opposite effects on AIP preference. Although physical health was negatively associated with AIP, age-friendly communities with compact, diverse, and accessible built environments have a positive impact on older adults’ physical health and therefore should be promoted.

**Translational Significance:** Developing countries, such as China, are facing the challenge of rapid population aging and ever-increasing care demand. This paper identified complex paths affecting older adults’ preference for a place to grow older at different scales; especially, we found that built environment intervention was associated with older adults’ self-rated health as well as the intention of independent living. The findings suggest the significance of social institutions in promoting aging in place (AIP) and inform urban planners and practitioners of the strategy for the development of age-friendly communities from the perspective of AIP.

China has the world’s largest aging population, and the aging of the national population is increasing at an unprecedented pace. By 2020, the number of people aged 60 and above in China reached more than 264 million, accounting for 18.7% of the total population of the country (National Statistics Bureau, 2021). Rapid population aging has raised various challenges for the welfare system, particularly the social care system for older adults. On the one hand, compared with many developed countries, the state has limited financial capability and resources to meet the care needs of a large aging population, and the formal care system in China is underdeveloped (Zhu & Walker, 2018). To address this issue, the Chinese central government launched a series of policies and aimed to build a social care system in which home-based care is the foundation, community-based care provides the necessary support, and institution-based care is supplementary (Shi & Hu, 2020). On the other hand, although connections and obligations between generations within Chinese families are still stronger than those in Western societies (Lu et al., 2021), the cultural traditions associated with filial piety have declined in China over time, and informal care provided by families has become less common (Cheng et al., 2015). Governmental policies, as well as changes in demography and social norms, have indicated the importance of maximizing older adults’ independence and autonomy, regardless of age, income, or ability level.

Aging in place (AIP) is considered to be an efficient strategy for improving the well-being and quality of life of older adults, and providing a solution to the shortage of workers in the formal caregiving workforce and the deterioration of informal support networks (Fernandez-Portero et al., 2017; Kasper et al., 2018). The concept of AIP has received substantial attention in the gerontological literature over the last 30 years. However, there is still no overall consensus on how AIP should be defined or on the factors and processes that influence it (Bigonnesse & Chaudhury, 2020). In the current study, we treat AIP as a residential and/or care choice that is opposite to institutionalization, and thus adopt Grimmer et al. ’s (2015) definition as follows: AIP is mostly about the opportunity for older people to remain in their own home without having to move to a long-term care facility. Research conducted in various countries has consistently suggested that most older people have a strong desire to age in their own homes for as long as possible, even in cases of health and physical limitations (Afacan, 2008; Costa-Font et al., 2009; Pani-Harreman et al., 2021; Wiles et al., 2012), so as to maintain a sense of self-reliance, self-management, and self-esteem (Milligan, 2009). On the other hand, some research works have also pointed out that the home context sometimes is over-romanticized as the ideal living environment, and AIP should not be a “one-stop” solution to later-life aspirations and needs (Hillcoat & Ogg, 2014). In many cases, older adults will consider institutionalization when opportunities for receiving good care are rated more highly than staying put, or when they do not want to be a burden for their families (Fernandez-Carro, 2016). In addition, it is necessary to be aware that older people’s relationship with the environment is more nuanced and complex than the duality of optimal and detrimental (Sim et al., 2012); similarly, the aging option is not a simple dualistic reaction of either remaining at home in community dwellings or relocation to institutional settings.

Although substantial empirical evidence has revealed the care and residential choices made by older adults, less attention has been paid to older adults’ ideal preferences, which may show disparity from reality and vary greatly because of personal attributes as well as the services and resources available to them. Moreover, most previous studies have considered that personal and/or community-level factors affect older adults’ choices, while neglecting the possible impacts of macro-scale factors. Using survey data from four cities located in China’s Yangtze River Delta, the current study aims to understand the association between the stated preferences for a place to grow older of older people and the factors potentially influencing these preferences at different scales from the city level, to the community level, and the individual level.

## Analytical Framework

We followed the ecological model of aging (Lawton, 1982; Lawton & Nahemow, 1973) to establish the theoretical framework of the current study. This model interprets older adults’ aging processes as an interaction between individual characteristics and environmental resources, and has been widely adopted in previous studies for the analysis of AIP and older people’s care and residential preferences (Choi, 2022; Greenfield, 2012; Shi & Hu, 2020). A core insight of this model in relation to aging is that individuals’ abilities and disabilities alter the extent to which environmental changes influence their functioning (Greenfield, 2012). Meanwhile, older people can proactively change their environments to meet their own needs and maintain independence (Golant, 2011). However, AIP fails when environmental pressure overwhelms the individual’s declining abilities. More recent models and concepts underlie the processes of person–environment (P-E) exchange from a differentiated perspective; for example, by linking the developmental models of aging well, researchers (Chaudury & Oswald, 2019; Wahl et al., 2012) integrate two P-E processes, namely behavioral-driven agency and experience-driven belonging, into existing P-E exchange. The former process refers to goal-directed behaviors related to making use of the objective physical–social environment whereas the latter refers to nongoal-oriented cognitive and emotional attachment to the environment. As people enter advanced old age, the processes of belonging increase in importance whereas the relevance of processes of agency decreases.

According to the ecological model, environmental factors influencing the aging process have overlapping layers (Sakar et al., 2016), which include the microsystem (i.e., the environmental context in individuals’ immediate surroundings), the mesosystem (i.e., the environmental settings that have an indirect impact on individuals), and the macrosystem (i.e., the broader system of values, laws, customs, and resources). The more distal settings influence individuals by altering aspects of progressively more proximal environments (Bronfenbrenner, 1979). The ecological model also distinguishes between the physical and social environments, and the latter comprises interpersonal relationships as well as broader social institutions and arrangements (Greenfield, 2012). Early studies had a pronounced focus on the social environment, with less emphasis on the physical environment (Wahl & Lang, 2006). Recently, the impact of the physical environment has drawn increasing attention; a widely used approach is addressing the physical features of and/or satisfaction with housing, which is the most proximal environmental factor for older adults. The contribution of key elements of public and community environments remained largely overlooked until the past decade (Wahl et al., 2012).

Findings in the existing literature suggest that social capital (e.g., interaction with neighbors, social activity participation, support from friends and community) fosters place attachment, and contributes to life satisfaction and intention for AIP (Han et al., 2021; Jiang et al., 2018; Wiles & Jayasinha, 2013). Regarding the physical environment, evidence suggests that home modification (Fausset et al., 2011), affordable housing (Peace et al., 2011), and universal and adaptive housing design (Demirkan, 2007; van Hoof et al., 2021) promote AIP. At the neighborhood level, several empirical studies reported that communities with better street connectivity, more diverse land use, and higher residential density were positively associated with AIP because these features were more supportive of walking and facilitated access to the necessary resources to meet older adults’ daily needs (Hartt et al., 2022; Rosso et al., 2013; Villanueva et al., 2014; Wang & Shepley, 2018). In addition, the availability of health services (e.g., clinics, pharmacies, hospitals), food resources (e.g., grocery stores, restaurants), green space, and sports and recreational facilities (e.g., sports centers, chess, and card rooms) also supported AIP (Dobner et al., 2016; van Dijk et al. 2015). Conversely, housing problems and adverse neighborhood environments (e.g., noise, traffic, crime) have been found to discourage AIP and decrease residential stability (Choi, 2022). Most previous research has focused on establishing the associations between the community-built environment and AIP through indicators such as physical activity, physical and/or mental health, life and/or housing satisfaction, and personal well-being; few studies have directly examined the effects of environmental context on preference for a place to grow older.

Individual factors that have been reported to contribute to AIP include demographic characteristics, socioeconomic status, functional capability, and needs. Findings regarding the impact of gender have been inconsistent; those with more family members, especially more children, are more willing to age in place (Park & Choi, 2021). Good mental and physical health status and a high level of satisfaction with daily life needs also promote AIP preference (Bosch-Farré et al., 2020). In contrast, older adults receiving a large pension from social security were more likely to choose mixed care (care services jointly provided by the community, the family, and government-subsidized care service providers) and state-based care (Lu et al., 2021). Other predictors of institutionalization include poor health condition, low support network, younger age, better education, and being urban citizens (as opposed to rural citizens; Park & Choi, 2021; Shi & Hu, 2020).

On the basis of the findings of previous studies, the current study further explored two main research questions: (1) What are older adults’ preferences for place to grow older in large cities in China, and are there any differences between different cities? (2) How are various individual and environmental factors at different scales, particularly factors related to the built environment of the city and community, interactively associated with older adults’ preference for place to grow older?

## Data and Methodology

### Study Area and Data Collection

**Figure 1.**
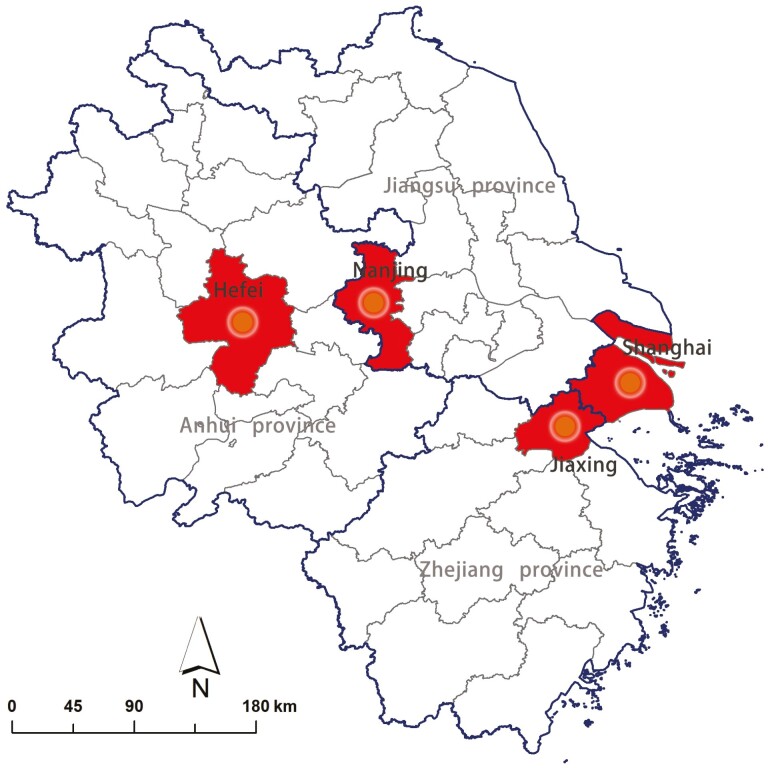
Study area and sample cities.

The Yangtze River Delta comprises one provincial-level city (Shanghai) and three provinces (Jiangsu, Zhejiang, and Anhui). This area has the largest urban agglomeration and is the most developed region in China. By 2021, the population of the Yangtze River Delta accounted for 16.7% of the population of Mainland China, producing 24.1% of the national gross domestic product (GDP). In the current study, we chose Shanghai and one city from each province in the Yangtze River Delta as our sample cities (Figure 1). The four selected cities belong to four different types in terms of population size, on the basis of the official standard announced by the Government (State Council, 2014). We evaluated city-level factors from four dimensions: demography, economy, built environment, and social welfare, each with three indicators (Table 1). Despite the great variation in population size, all four cities are highly urbanized with high proportions of aging population (from 15.26% to 23.40%). In terms of economy, Nanjing, the capital city of Jiangsu province, has the highest GDP per capita of about 25,000 US dollars whereas Shanghai has the highest employee salary and government expenditure; the other two cities share a similar and lower economic status. There are three major types of medical care in China: urban employee medical care, urban resident medical care, and new rural cooperative medical care; among them, urban employee medical care is jointly paid by the employer and employee with a higher premium and provides better coverage. As shown in Table 1, in Shanghai, nearly two thirds of the population have registered with the urban employee medical care, whereas this proportion in Hefei is merely 25.15%.

**Table 1.** Profile of the Sample Cities (Yearbook of Chinese City, 2021)

Variable	Shanghai	Nanjing	Hefei	Jiaxing
Population (10,000 person)	2,488	932	937	541
Urbanization ratio (%)	89.3	86.8	82.3	71.3
Proportion of aging population (%; 60 and above)	23.40	19.36	15.26	18.70
GDP per capita (U.S. dollar)	23,969	24,511	16,681	15,776
Average annual salary for employee in urban nonprivate sector (U.S. dollar)	26,874	21,232	16,126	16,556
Expenditure in local government’s public budget per capita (U.S. dollar)	5,010	2,896	1,912	2,025
Green land per capita (m^2^)	66.16	100.33	21.63	11.36
Road network density (km/km^2^)	2.04	1.49	1.75	2.11
Annual average concentration of PM2.5 (μg/m^3^)	32	31	36	28
Number of licensed doctors per 10,000 people	31.50	40.58	31.00	28.32
Ratio of population with pension insurance (%)	64.98	36.22	30.71	36.48
Ratio of population with urban employee medical care (%)	63.84	51.90	25.15	47.16

*Notes*: GDP = gross domestic product; PM2.5 = particulate matter 2.5.

A questionnaire survey was conducted in the sample cities in December 2020, with assistance from a survey company (Better Consulting, http://www.btmr.cn/). A pilot survey was conducted in advance by interviewing 20 older adults to make sure the survey questions and options were reasonable and understandable. The study used a two-stage sampling approach. In the first stage, the investigators first identified one public space in each urban district of the sample cities in which older adults often gathered (e.g., park, supermarket), and 22 venues were then selected. In each venue, the investigators then randomly recruited participants; only people aged 60 and above who had been living at their current address for more than half a year were included in the survey. Participants proceeded to answer the questions after agreeing to take part in the survey. A total of 827 completed questionnaires were collected (Shanghai: 219; Nanjing: 204; Hefei: 204; Jiaxing: 202), with a response rate of 32.3%. Diversity in the sample in terms of age, gender, socioeconomic status, self-rated health, and residential location was met. Cronbach’s α and factor analysis showed the data had good reliability and validity. The home address of each respondent was recorded for the measurement of the home and community features (HCF); data regarding the built environment were crawled from Anjuke, a real-estate-trading website (www.anjuke.com); Worldpop, an open-access population database (www.worldpop.org); Gaode map (lbs.amap.com); and Open Street Map (www.openstreetmap.org). Normalized difference vegetation index (NDVI) was measured on the basis of remote sensing data downloaded from Geospatial Data Cloud (www.gscloud.cn).

### Methods and Hypotheses

On the basis of the analytical framework established earlier, we measured the interaction between AIP preference (interchangeably used with “preference for place to grow older” hereafter), various individual characteristics, and social and physical environmental factors. In the existing literature, the concept of AIP preference can be operationalized as a continuous variable (e.g., length of years a person intends to continue to live in the current place; Lehning et al., 2022), a dummy variable (e.g., stay in the community vs move to a nursing home; Lum et al., 2016), or a categorical variable (e.g., one’s own home, a nursing home, or a relative’s home; Costa-Font et al., 2009). With reference to the literature and options available in Chinese cities, AIP preference in this paper was assessed using the question, “Ideally, which of the following places would you prefer to live in as you get older?” with four response options: my own home, my children’s home, community daycare center, and long-term care agency. Individual characteristics obtained from the survey included age, gender, type of household registration (*hukou*), medical insurance status, length of residence, family structure, and self-reported physical health. We used a modified short Warwick–Edinburgh mental well-being scale to evaluate respondents’ mental health (Table 2).

**Table 2.** Questions to Evaluate the Mental Health of the Surveyed Older Adults

Statement (for your experience over the last 2 weeks)	Never	Rarely	Sometime	Often	All the time
Q1 I’ve been feeling optimistic about the future	—	0.7%	4.5%	29.1%	65.7%
Q2 I’ve been feeling relaxed	—	0.7%	3.1%	21.0%	75.1%
Q3 I’ve been dealing with family problems well	—	1.0%	2.8%	16.6%	79.7%
Q4 I’ve been thinking clearly	—	0.4%	1.1%	17.5%	81.0%
Q5 I’ve been feeling close to my children and other people	0.01%	0.4%	4.1%	22.6%	72.8%
Q6 I’ve been feeling lonely	58.6%	34.0%	6.2%	0.8%	0.4%

We measured the social environment by asking respondents about the frequency with which they participated in a range of common social activities for older adults in China, including gathering with friends, participating in group exercise, playing board and card games, and traveling with other people. Because most urban-dwelling citizens in China live in collective apartments, the home features considered in this study included housing price, floor area ratio, and green space ratio of the apartments. Community-level built environments were measured using ESRI ArcGIS 10.3. Respondents’ home addresses were located on the city map according to their coordinates. For each home address, a 1-km buffer was generated; road network density (km/km^2^), population density, land-use mix, and NDVI within the buffer were measured. Among them, land-use mix was calculated using the following formula:


$$\begin{array}{l} \;M_{i}=\frac{-\overset{k}{\underset{k\;=\;1}{\sum }}P_{k,i}\;ln(P_{k,i})}{ln(-K,i)} \end{array}$$

where *M*_*i*_ indicates the land-use mix of respondent *i*’s community (range 0–1), *K* is the number of point of interest (POI) types within the buffer, and *P*_*k,i*_ is the proportion of type-*k* POI among all of the POI types. We also measured the density of medical resources, food outlets, green spaces, sports and recreation facilities, and neighborhood facilities within each buffer on the basis of POI data. To make these built environment variables comparable, they were all standardized using the *Z*-score method. Because the existing literature suggested that perceived built environments might also have impacts on older people’s behavioral decisions (Pan et al., 2020; Tropped et al., 2017), we also evaluated respondents’ perceptions regarding their communities’ built environment in the questionnaire (Table 3).

**Table 3.** Questions to Evaluate the Perceived Community-Built Environment of the Older Adults

Statement	Strongly agree	Agree	Disagree	Strongly disagree
Q1 It is convenient for me to go to see doctors	63.7%	31.4%	4.0%	0.80%
Q2 It is convenient for me to do daily shopping	81.4%	17.5%	1.1%	—
Q3 It is convenient for me to take public transit	80.9%	17.3%	1.7%	0.01%
Q4 It is convenient for me to visit park	66.1%	23.7%	9.3%	0.80%

We first ran a multinomial logistic regression model with AIP preference as the dependent variable (results not reported here), and found that although individual characteristics and social environmental factors were significantly associated with AIP preference, most physical environmental factors did not have statistically significant effects. However, we found that many built environment variables were strongly correlated with self-reported health status in Pearson’s correlation analyses, we speculated that there might be some indirect effects of the physical environment on AIP preferences. Therefore, we used structural equation modeling (SEM) in the subsequent analysis and constructed the following hypotheses:

H1: On the basis of the interrelationships between proximal and distal environments in ecological frameworks (Greenfield, 2012), we expect city-level environment affects AIP preference both directly and indirectly via the mediation effect on community-level environment.

H2: On the basis of ecological models of aging and the empirical evidence on the association between built environments and public health (Choi, 2022; Hartt et al., 2022; Wang & Shepley, 2018), we expect respondents’ perceptions regarding the community’s built environment have a positive impact on their AIP preference via the mediation effect on personal health status.

H3: Similar to Hypothesis 2, we expect objectively measured built environments of respondents’ communities to have a positive impact on their AIP preference via the mediation effect on personal health status.

H4: According to the ecological frameworks, social environment and individual characteristics are both key components in the processes of P-E exchange (Chaudury & Oswald, 2019; Wahl et al., 2012). Therefore, we expect respondents’ social environment to have a positive impact on their AIP preference.

H5: Respondents’ individual characteristics will affect their AIP preference.

## Results

### Descriptive Analysis

As shown in Table 4, respondents’ average age was 69.5 years old, with similar proportions of male and female respondents. The majority of respondents were local residents and had lived in the current community for an average period of 47.7 years. Most respondents were registered with urban employee medical care. The respondents tended to have a positive or neutral assessment of their physical health, and the self-assessment of their mental health was better than the self-assessment of their physical health (Table 2). Regarding preference for place to grow older, approximately two thirds of respondents expressed a preference for living in their own home, indicating a strong AIP preference. The proportions of respondents choosing to live with their children and those choosing to live in a long-term care institution were similar. The least preferred option was living in a community daycare center. More than half (52.6%) of the respondents needed the community to provide services in daily life (e.g., food catering and cultural activities), whereas psychological assistance was considered to be the least needed service. Table 5 shows the results for HCF and the availability of community services. Substantial disparities in characteristics of the built environment were exhibited between different communities, especially for road network density, the distribution of green space, and neighborhood facilities.

**Table 4.** Descriptive Information on the Respondents

Variable	Mean (*SD*)	%
Age (in years)	69.5 (6.7)	
Gender		
1. Male		50.9
2. Female		49.1
Household registration		
1. Local		84.0
2. Nonlocal		16.0
Medical insurance		
1. Urban employee medical care		73.5
2. Other		26.5
Length of residence (in years)	47.7 (22.9)	
Self-reported physical health (1: very good–5: very poor)	2.16 (0.86)	
Preference for place to grow older		
1. My own home		65.1
2. My children’s home		15.7
3. Community daycare center		3.70
4. Long-term care agency		15.5
Most (least) needed care service		
1. Medical attendance		46.8 (16.8)
2. Service in daily life		52.6 (40.7)
3. Psychological assistance		0.60 (42.6)

*Note: SD* = standard deviation.

**Table 5.** Descriptive Information on the Community-Built Environments

Variable	Max.	Min.	Mean	*SD*
Road network density (km/km^2^)	38.00	0.00	5.99	9.42
Land-use mix	0.98	0.00	0.80	0.18
Normalized difference vegetation index (NDVI)	0.99	0.00	0.27	0.28
Population density (/km^2^)	44,944	813	14,163	10,196
Housing price of the apartment (dollar/m^2^)	23,513	462	6,253	4,872
Floor area ratio (FAR) of the apartment	3.60	0.24	1.82	0.52
Green coverage of the apartment	0.60	0.10	0.33	0.09
Density of medical facility (including hospital, clinic, drug store; /km^2^)	288	0	93	60
Density of food outlets (including supermarket, restaurant, grocery/convenience store; /km^2^)	1,631	0	408	257
Density of parks and open space (/km^2^)	168	0	17	25
Density of sports and recreational facility (/km^2^)	415	1	103	71
Density of neighborhood facility (including laundry, barber shop, bank, post office, etc.; /km^2^)	2,277	0	213	403

*Note*: *SD* = standard deviation.

### Association Between AIP Preference and City-Level Factors

**Figure 2.**
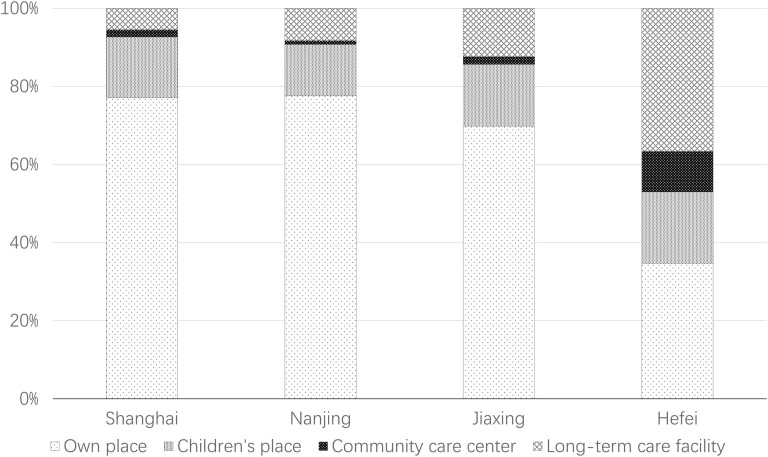
Preference for place to grow older by cities.

Preference for place to grow older of the respondents from different cities has significant variance (Pearson’s χ^2^ = 153.227, *p* < .000; Figure 2). In Shanghai and Nanjing, approximately 77% of respondents expressed a preference for staying in their own home as they get older, whereas this proportion was only 34.7% in Hefei. In contrast, respondents from Hefei had the highest proportion of respondents (36.6%) expressing a preference for living in a long-term facility. We carried out a correlation analysis between preferences and city-level factors (Table 6); the Eta^2^ and *p* values of the analysis of variance revealed that the proportion of the population registered with urban employee medical care was the factor most strongly associated with preferences; as explained earlier, urban employee medical care is more advantaged compared with other types of medical care, and its higher registration rate indicates a city has more capacity and resources to provide daily health support to its residents, which may increase older people’s AIP preference. GDP per capita and annual average concentration of particulate matter 2.5 also had strong associations with preferences. In contrast, the urbanization ratio, proportion of aging population, road network density, and number of doctors per 10,000 people were nonsignificantly or weakly associated with preferences. The other five factors had a medium-strength association with preferences.

**Table 6.** Correlation Between Preference for Place to Grow Older and City-Level Factors

Variable	Eta^2^	*p* Value
Population	0.08	.000
Urbanization ratio	0.023	.000
Proportion of aging population (60 and above)	0.007	.143
GDP per capita	0.13	.000
Average annual salary for employee in urban nonprivate sector	0.076	.000
Expenditure in local government’s public budget per capita	0.066	.000
Green land per capita	0.061	.000
Road network density	0.005	.235
Annual average concentration of PM2.5	0.107	.000
Number of licensed doctors per 10,000 people	0.019	.001
Ratio of population with pension insurance	0.06	.000
Ratio of population with urban employee medical care	0.163	.000

*Notes*: GDP = gross domestic product; PM2.5 = particulate matter 2.5.

### Association Between AIP Preference and Community- and Individual-Level Factors

**Figure 3.**
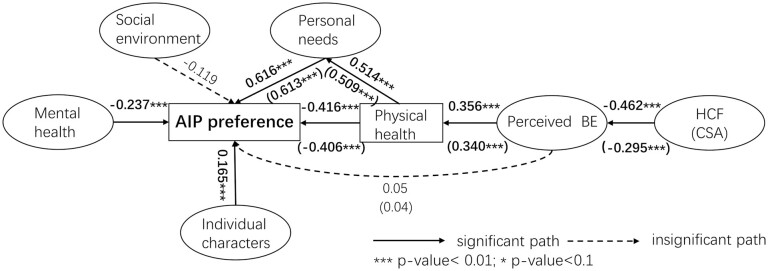
Results of the structural model. AIP = aging in place; BE = built environment; CSA = community service availability; HCF = home and community features

We used Mplus7.4 (Muthén and Muthén, 2015) to carry out the SEM analysis, because this software can deal with categorical variables. People who hoped to live in their own home or their children’s home were considered to have a stronger AIP preference (coded as 1) whereas people who hoped to live in a community daycare center or long-term care facility were considered to have a stronger institutionalization preference (coded as 2). Thus, a binary variable was created to represent the preference for place to grow older. On the basis of pretests using component factor analysis, the measurement model was constructed (Table 7). Mental health, the social environment, and the perceived built environment were represented by the corresponding questions from the questionnaire. Personal characteristics were composed of five observed variables; among them, Age and Length of residence were transformed to categorical variables to be consistent with the other three variables. Personal care needs were reflected by the respondents’ most needed service, least needed service, and whether they agreed that the community had provided sufficient services for older people. The community-built environment was measured by two latent variables: HCF and community service availability (CSA), which were represented by seven and five observed variables, respectively. Several structure models were tested, and the final models and standardized coefficients were reported in Figure 3. Because HCF and CSA were strongly correlated, they were entered into the model separately, and two models were kept. Model diagnosis (Table 8) showed that the goodness-of-fit values for both models were acceptable.

**Table 7.** Results of the Measurement Model

Latent variable	Observed variable	Model 1 estimate	Model 2 estimate
Mental health	Q1 I’ve been feeling optimistic about the future	0.425***	0.425***
	Q2 I’ve been feeling relaxed	0.624***	0.624***
	Q3 I’ve been dealing with family problems well	0.611***	0.611***
	Q4 I’ve been thinking clearly	0.622***	0.622***
	Q5 I’ve been feeling close to my children and other people	0.526***	0.526***
	Q6 I’ve been feeling lonely	−0.468***	−0.468***
Individual characters	Gender	0.121**	0.121**
	Age	−0.266***	−0.266***
	Household registration	0.981***	0.981***
	Medical insurance	0.757***	0.757***
	Length of residence	−0.796***	−0.796***
Personal needs	Provision of service	0.566***	0.546***
	Most needed service	−0.406***	−0.393***
	Least needed service	0.258***	0.283***
Social environment	Gathering with friends	0.622***	0.622***
	Group exercise	0.696***	0.696***
	Playing card games	0.389***	0.389***
	Traveling with others	0.573***	0.573***
Perceived built environment (BE)	Q1 It is convenient for me to go to see doctors	0.605***	0.589***
	Q2 It is convenient for me to do daily shopping	0.525***	0.503***
	Q3 It is convenient for me to take public transit	0.641***	0.621***
	Q4 It is convenient for me to visit park	0.802***	0.868***
Home and community features (HCF)	Road network density	0.356***	
	Land-use mix	0.476***	
	Normalized difference vegetation index (NDVI)	0.189**	
	Population density	0.458***	
	Housing price	0.361***	
	Floor area ratio (FAR)	0.047	
	Green space ratio	−0.115*	
Community service availability (CSA)	Medical facility density		0.858***
	Food outlet density		0.879***
	Open space density		0.799 ***
	Sports/recreational facility density		0.793***
	Neighborhood facility density		0.818***

***p < .01. **p < .05. *p < .1.

**Table 8.** Model Fit Information

	χ^2^/DF	Comparative fit index	Tucker–Lewis index	Root-mean-square error of approximation
Model 1 (with HCF)	3.36	0.745	0.722	0.054
Model 2 (with CSA)	3.19	0.840	0.825	0.051
Reference	<5	>0.7	>0.7	<0.08

*Notes*: CSA = community service availability; HCF = home and community features.

For the measurement model, all of the observed variables had significant associations with the latent variables, except housing price and HCF. For the structural model of Model 1, mental health and individual characteristics had direct effects on AIP preference, and the community social environment had no significant effect (*p* = .137). Physical health had a direct, negative association and an indirect, positive association with AIP preference via the mediation effect of personal needs; the total effect of physical health was negative (−0.416 + 0.616 × 0.514 = −0.099). Perceived built environment of the community had no direct impact on AIP preference (*p* = .42), but showed an indirect effect via the mediation of physical health (−0.416 × 0.356 = −0.148). The objectively measured built environment significantly influenced AIP preference via a chain effect (−0.416 × 0.356 × (−0.462) = 0.068). Model 2 had a very similar structure to Model 1, only with a much lower influence of the objectively measured built environment on AIP preference (−0.406 × 0.340 × (−0.295) = 0.041).

## Discussion

In accordance with most existing literature regarding AIP (Pani-Harreman et al., 2021; Wiles et al., 2012; Zhong et al., 2022), the older adults in our study expressed a dominant preference for AIP. In several previous studies conducted in China, urban residents were reported to exhibit greater acceptance of institutionalization than rural residents (Lu et al., 2021; Shi & Hu, 2020). In the current study, we further distinguished preference for place to grow older among urban residents: namely, our results revealed that older adults from more developed cities expressed stronger preferences for AIP compared with older adults from less developed cities.

Regarding the factors influencing AIP, we found that at the city level, both the social and physical environments affected AIP preference, and the former had a stronger impact than the latter. These macro-level factors have received little attention in previous studies. Generally, higher average income, better established social and medical insurance systems, more green space, and better air quality increase older adults’ preference for AIP. On the basis of the ecological model, city-level factors, which are more distal, would be expected to influence individuals’ preferences through more proximal community-level factors (Greenfield, 2012). However, when we tried to introduce city-level factors into SEM analysis, the model cannot converge; as a result, we were not able to verify this mediation effect in our model, Hypothesis 1 was partially supported.

At the community level, the physical environment but not the social environment affected AIP preference. HCF had a greater influence on AIP preference than the availability of community services. More age-friendly community-built environments (in terms of higher density, more land-use diversity, and better access to various services) were positively associated with better-perceived community environment, as reported in previous studies (Ma et al., 2014). Further, the positive perceptions would positively affect self-reported physical health (Barnett et al., 2017; Liu et al., 2018; Wang et al., 2019). A novel finding in the current study was that physical health had a negative direct effect on AIP preference, but a positive indirect effect via influencing older adults’ care needs, and the total effect was negative. The findings of previous studies in Europe (Corneliusson et al., 2019; Fernandez-Carro, 2016) and the United States (Choi, 2022) have largely suggested a positive association between health status and AIP preference. Studies conducted in China (Lu et al., 2021) and South Korea (Park & Choi, 2021) examined this relationship and reported nonsignificant results. Regarding the association between the community-built environment and AIP, as discussed earlier, most previous studies have used physical activity, life and/or housing satisfaction, and personal well-being as indicators for AIP. To the best of our knowledge, only one previous study has reported a positive association between the direct measurement of AIP (measured by the length of residence) and the community-built environment (Wang & Shepley, 2018). Our result suggested that improving the community-built environment may reduce the incentive for AIP, which appears to be contradictory with the ecological model. This finding is due to the negative association between physical health, the mediate variable, and AIP preference. We have attempted to explain this paradox as follows: “the ideal place to grow older” had rich connotations for the older adults, and it can be decomposed into at least two preferences, namely the residential preference and preference for place of care (Fernandez-Carro, 2016), as revealed by the two paths in our SEM analysis. Regarding the preference for place of care, older adults with worse health status had a stronger desire for institutionalization because being at a care facility can enable individuals to have their care needs better met compared with being at home, as suggested by the positive mediation effect of personal care needs in the model. This was a process of behavioral-driven agency (Wahl et al., 2012). On the other hand, as the health of older adults declined, they developed a stronger attachment to home and family, which led to a stronger AIP preference reflected in their residential preference. This was a process of experience-driven belonging (Wahl et al., 2012). When the residential preference for AIP outweighed the care preference for institutionalization, which was in line with the life-course perspective of the interaction of belonging and agency, the total effect would be negative; therefore, Hypotheses 2 and 3 were not supported.

Inconsistent with the results of previous studies (Bigonnesse & Chaudhury, 2020; Lehning, 2015), we found that although the social environment had a tendency to positively affect AIP preferences, the effect was not statistically significant. We attribute this finding to the questions for evaluating the social environment in the survey not being sufficiently sophisticated, and not fully reflecting our respondents’ social capital. Hypothesis 4 was not supported by the current findings.

At the individual level, demographic and socioeconomic status were significantly associated with AIP preference. Our results revealed that male compared with female respondents, and younger compared with older respondents, had stronger preferences for AIP. Additionally, older adults with better socioeconomic status had stronger incentives for AIP, which was also inconsistent with previous studies (Bosch-Farré et al., 2020; Park & Choi, 2021). This discrepancy may have resulted from socioeconomic status being evaluated differently in our study than in previous studies; instead of using income, we used *hukou* and type of pension and medical insurance as indicators. Hypothesis 5 was supported by the current findings.

One strength of the current study is the multiscale and multivariate approach used for analyzing the stated preference of older adults for place to grow older. To the best of our knowledge, the current study is the first city-level comparison on this topic. Additionally, the SEM method revealed some unique findings that have not been reported previously. Another strength of this paper is its comprehensive consideration of the perceived and objectively measured community-built environment and the effort to elucidate its association with AIP preference. The current study involved the following limitations: First, because of the sampling method and the focus on the community-built environment, we did not include older adults with disabilities or those who were living in a care facility, which may have led to an exaggeration of the reported AIP preference. Second, some inconsistencies between the current findings and the existing literature were revealed, and the explanation for these discrepancies remains to be verified; to further explore this issue, more survey data are needed. This issue should be addressed in the follow-up studies in the future.

## Conclusion

The preference for a place to grow older of older adults is influenced by complex and diverse factors. Although our empirical study supports the notion that AIP is the preferred option for older adults in most cases, we found that preferences varied significantly across different cities. Older people living in more developed regions with better socioeconomic and mental health status expressed stronger AIP preference. For some older people, institutionalization is appealing in terms of satisfying care needs; however, older Chinese adults become increasingly willing to live in their original home as they become physically weaker. At the city level, the social environment had a stronger influence than the physical environment on AIP, and this pattern was reversed at the community level.

The current findings suggest that policies to address broad social institutions and arrangements (e.g., the pension insurance and medical care systems) and promote the development of age-friendly communities will be effective in meeting the challenges posed by rapid population aging in societies like China. Policy-makers and family members should pay attention to the mental health status of the older adults, which is positively associated with AIP preference according to our research but is largely neglected currently compared with physical health. The findings also inform urban planners and practitioners that some near-term practical strategies such as providing more daily facilities and services in communities with a higher proportion of older adults should be prioritized to improve older adults’ perception of their living environment. In the long run, community-built environments with compact, diverse land use and dense road network should be encouraged as they were found to have positive impacts on older adults’ self-reported physical health and thus reduce their need for formal care services from care agencies.

## Data Availability

The data sets used and/or analysis during the current study are available from the corresponding author on reasonable request. The study reported in the manuscript was not preregistered.
